# Infant flow biphasic nasal continuous positive airway pressure (BP- NCPAP) vs. infant flow NCPAP for the facilitation of extubation in infants' ≤ 1,250 grams: a randomized controlled trial

**DOI:** 10.1186/1471-2431-12-43

**Published:** 2012-04-04

**Authors:** Karel O'Brien, Craig Campbell, Leanne Brown, Lisa Wenger, Vibhuti Shah

**Affiliations:** 1 Department of Paediatrics, Mount Sinai Hospital, Toronto, Canada; 2 Department of Paediatrics, University of Toronto, Toronto, Canada; 3 Department of Respiratory Therapy, The Hospital for Sick Children, Toronto, Canada; 4 Department of Respiratory Therapy, Mount Sinai Hospital, Toronto, Canada

**Keywords:** Infant-newborn, Non-invasive ventilation, Continuous positive airway pressure, Extubation failure

## Abstract

**Abstract:**

**Background**

The use of mechanical ventilation is associated with lung injury in preterm infants and therefore the goal is to avoid or minimize its use. To date there is very little consensus on what is considered the "best non-invasive ventilation mode" to be used post-extubation. The objective of this study was to compare the effectiveness of biphasic nasal continuous positive airway pressure (BP-NCPAP) vs. NCPAP in facilitating sustained extubation in infants ≤ 1,250 grams.

**Methods**

We performed a randomized controlled trial of BP-NCPAP vs. NCPAP in infants ≤ 1,250 grams extubated for the first time following mechanical ventilation since birth. Infants were extubated using preset criteria or at the discretion of the attending neonatologist. The primary outcome was the incidence of sustained extubation for 7 days. Secondary outcomes included incidence of adverse events and short-term neonatal outcomes.

**Results**

Sixty-seven infants received BP-NCPAP and 69 NCPAP. Baseline characteristics were similar between groups. The trial was stopped early due to increased use of non-invasive ventilation from birth, falling short of our calculated sample size of 141 infants per group. The incidence of sustained extubation was not statistically different between the BP-NCPAP vs. NCPAP group (67% vs. 58%, P = 0.27). The incidence of adverse events and short-term neonatal outcomes were similar between the two groups (P > 0.05) except for retinopathy of prematurity which was noted to be higher (P = 0.02) in the BP-NCPAP group.

**Conclusions**

Biphasic NCPAP may be used to assist in weaning from mechanical ventilation. The effectiveness and safety of BP-NCPAP compared to NCPAP needs to be confirmed in a large multi-center trial as our study conclusions are limited by inadequate sample size.

**Clinical Trials Registration #**

NCT00308789

**Source of support:**

Grant # 06-06, Physicians Services Incorporated Foundation, Toronto, Canada. Summit technologies Inc. provided additional NCPAP systems and an unrestricted educational grant.

Abstract presented at The Society for Pediatric Research Meeting, Baltimore, USA, May 2nd-5^th^, 2009 and Canadian Paediatric Society Meeting, June 23^rd^-29^th^, Ottawa, 2009.

## Background

With advances in neonatal care, > 85% of infants with birth weight < 1,500 grams now survive [1,2]. Parallel to this improved survival is the increase in the incidence of bronchopulmonary dysplasia (BPD). In 2001, the National Institute of Child Health and Human Development Neonatal Research Network reported an incidence of BPD of up to 40% in infants < 1,000 grams [1] while the incidence of BPD was reported to be 21.4% (inter-quartile range 12.5%, 30.6%) for infants born between 501-1,500 grams in 750 neonatal intensive care units (NICUs) participating in the Vermont Oxford Network for the year 2008 [3]. Similarly in Europe, the incidence of BPD was 19.6% for the year 2006 among 60 NICUs participating in the EuroNeoNet [4]. Recently in 2010, Finer et al reported the incidence of BPD to be as high as 44% for infants born between 24^0/7 ^to 27^6/7 ^weeks gestation [5].

Bronchopulmonary dysplasia is a multi-factorial condition with interplay of antenatal, genetic and environmental factors. Central to its pathogenesis are lung immaturity and the use of mechanical ventilation [6]. It has been hypothesized that earlier extubation and use of nasal continuous positive airway pressure (NCPAP) may decrease lung inflammation and reduce the incidence of BPD [7]. It may also reduce ventilator-associated pneumonia and necrotizing tracheitis. Further, baboon studies suggest that the early use of CPAP may mitigate the decreased brain growth and cerebral neuropathologies seen in preterm infants who require ventilation [8]. Supporting evidence in human neonates comes from the results of the caffeine for apnea of prematurity trial where the investigators demonstrated that a reduction in the duration of positive pressure ventilation (of 1 week) [9] through an endotracheal tube was associated with an improved rate of survival without neuro-developmental disability (reduced rate of cerebral palsy and cognitive delay) [10].

The Infant Flow™ System (Viasys Healthcare Inc, Yorba Linda, CA, USA) used in our study is the most widely utilized variable flow device. It uses high velocity jet flows that can entrain gas on demand during inspiration and therefore keep the CPAP level constant. On exhalation the design of the nasal prongs results in some of the fresh gas being shunted away through an expiratory outlet rather than continuing to the nares reducing the expiratory work [11-14]. In contrast to regular NCPAP which provides a continuous distending pressure, biphasic NCPAP (BP-NCPAP) cycles between upper and lower (baseline) level pressures as determined by the following four parameters a) lower CPAP level b) upper CPAP level c) time at upper level and d) rate (cycles/minute at upper level). Theoretically, functional residual capacity is recruited by the upper CPAP level and maintained with the lower baseline CPAP level, thus decreasing the work of breathing. To date there have been no studies comparing the use of these two modes of non-invasive ventilation in preterm infants to facilitate sustained extubation following an initial period of intubation and positive pressure ventilation at birth.

The primary goal of this study was to compare the effectiveness of BP-NCPAP vs. NCPAP using the Infant Flow^® ^SiPAP™ Viasys Healthcare Inc. system in facilitating sustained extubation in preterm infants ≤ 1,250 grams. The secondary goals were to compare the adverse events and short-term neonatal morbidities between the two groups.

## Methods

In this randomized controlled trial we included intubated infants with birth weight ≤ 1,250 grams. Infants with congenital abnormalities of the upper airway tract, acquired nasal septum injury and major congenital or chromosomal abnormalities were excluded. The study was conducted at a tertiary care NICU, Mount Sinai Hospital, Toronto, Ontario, Canada, during the period from April 2006 to November 2008.

Parents of eligible infants were approached for participation in the trial and written informed consent was obtained prior to extubation. A marker was then placed at the bedside of eligible infants whose parents had given consent. Randomization cards were generated using a computer generated random numbers list. The cards were sealed in sequentially marked opaque envelopes and opened immediately prior to the first extubation. Infants were randomized to one of two groups: BP-NCPAP or NCPAP delivered by the Infant Flow^® ^SiPAP™ (Viasys Healthcare, Yorba Linda, CA, USA). The assigned mode of support was continued until the infant was ready to be placed in room air or supplemental oxygen. The study was approved by the local Research Ethics Board.

Preset criteria were used to guide extubation using a consensus approach amongst neonatologists in our NICU. For conventional ventilation the criteria included: a ventilator rate of < 20 breaths per minute (bpm), peak inspiratory pressure (PIP) ≤ 16 cm H_2_O and fractional inspired oxygen (FiO_2_) of ≤ 0.35. For high frequency ventilation the criteria were: frequency of 9-13 Hz, amplitude < 20 percent, mean airway pressure (MAP) of ≤ 8 cm H_2_O and FiO_2 _≤ 0.35. Once an infant reached these preset criteria, the medical team was approached for consideration of extubation. In the event of accidental extubation in eligible consented infants, face mask CPAP was applied for no more than 15 minutes until a decision was made either to reintubate based on the clinical condition or to randomize to the study group. All infants had the appropriate bonnet, nasal prong interface and Cannulaide^® ^(Beevers Manufacturing Inc., McMinnville, OR, USA) applied.

In the BP-NCPAP arm the respiratory rate was set at 20 bpm with an inspiratory time of 1.0 second. The upper level of CPAP was set 3 cm above the lower (baseline) level of CPAP. In both modes the lowest baseline CPAP was set at 5 cm H_2_O and the CPAP was titrated according to the infant's FiO_2 _needs based on an algorithm (Table 1). Neither mode of NCPAP was synchronised with the infant's respiratory effort. Weaning in both groups was left at the discretion of the attending neonatologist. If the infant remained clinically stable in FiO_2 _≤ 0.25 with no evidence of increased work of breathing and/or apnea of prematurity, then attempt was made to trial off CPAP.

**Table 1:** Guidelines for use of biphasic nasal continuous positive airway pressure (BP-NCPAP) and nasal continuous positive airway pressure (NCPAP)

Settings for BP- NCPAP	FiO_2_* (%)	< 0.30	0.30 - 0.50	> 0.50
	
	Upper CPAP (cm H_2_O)	8	9	10
	
	Lower CPAP (cm H_2_O)	5	6	7
**Settings for NCPAP**	FiO_2 _(%)	< 0.30	0.30 - 0.50	> 0.50
	
	CPAP (cm H_2_O)	5	6	7

*FiO_2 _= Fraction of inspired oxygen

Criteria for reintubation included: presence of severe apnea (defined as need for positive pressure ventilation), ≥ 4 minor apneic episodes per hour requiring moderate stimulation, required supplemental oxygen of > 60% to maintain oxygen saturation > 88%, developed uncompensated respiratory acidosis (defined a pH < 7.25) or a combination of the above. Apnea was defined as cessation of respiration for > 20 seconds or a shorter pause if associated with bradycardia (heart rate < 100 beats per minute) or desaturation (< 85%). Reintubation was also allowed at the discretion of the attending medical team for other reasons, e.g., concerns regarding sepsis. Data were collected for the duration of their in-hospital stay. Other medical therapy and interventions were provided at the discretion of the medical team.

In our unit, caffeine is usually commenced in the first week of life even if the infant requires positive pressure ventilation via endotracheal tube. A loading dose of 10 mg/kg followed by maintenance dose of 2.5 mg/kg is administered within 24-36 hours. Based on the clinical response the maximum dose of maintenance caffeine used is 5 mg/kg.

Data were collected on maternal characteristics including age, gravidity, parity, pregnancy induced hypertension, essential hypertension, preterm prolonged rupture of the membranes (> 18 hours), antenatal steroids (complete and partial course), and clinical and histological diagnosis of chorioamnionitis from maternal health records and placental pathology.

The primary outcome was the incidence of sustained extubation for 7 days. Secondary outcomes included incidence of adverse events such as: nasal septal injury/erythema, eyelid edema, abdominal distension, feeding intolerance and pneumothorax. Nasal septal injury/erythema and eyelid edema were monitored and recorded every 4 hours by the respiratory therapists and the nursing staff. Data on feeding intolerance (defined as aspirates of ≥ 30% of a single feed administered) and abdominal distension (defined as > 10% increase in abdominal girth) were recorded by the nursing staff every 4 hours and/or prompted by clinical concerns. Data on the other clinical outcomes including the incidence of BPD [oxygen dependency at 36 weeks post menstrual age (PMA)], patent ductus arteriosus (PDA) (diagnosed clinically or by ECHO and treated with indomethacin ± surgery), necrotizing enterocolitis (NEC) (Bell's stage 2 or greater) [15], grade 3/4 intraventricular hemorrhage (IVH) [16] or periventricular leucomalacia (PVL) and retinopathy of prematurity (ROP) were abstracted from the chart. Retinopathy of prematurity was classified according to the international classification [17]. Infants who died were excluded from the analysis for ROP and for BPD if they died before they reached 36 weeks PMA. In our unit, PDA is treated pharmacologically (with indomethacin) based on the presence of clinical symptoms and signs. Prior to administration of a second course of indomethacin or referral for surgical ligation, infants undergo echocardiography. Both caregivers administering the interventions and research assistants were not blinded to the group assignment.

The sample size calculation was based on the results obtained from a previous study that compared the rate of sustained extubation using NCPAP vs. high flow oxygen in our unit. The rate of sustained extubation with NCPAP was 85% [18]. To demonstrate a clinically significant increase in the rate of sustained extubation by 10% between groups (i.e. from 85% to 93.5%) with 80% power and an alpha value of 0.05, we estimated a sample size of 141 patients in each arm for a total of 282 patients.

The analysis was performed using the intention-to-treat principle. Baseline maternal and infant characteristics and outcomes of the infants randomized to both modes were compared using χ^2 ^test for categorical data and Student's *t *test for continuous data. The Wilcoxon rank sum test was used to compare continuous data with highly skewed distributions. All reported P values are two sided. A planned secondary analysis examined the predictors of successful extubation using multivariate logistic regression to control for possible confounders including birth weight, sex, age at the time of first extubation, accidental extubation and use of antenatal steroids. All statistical analyses were performed using the computer program Statistical Package for the Social Sciences v.12™ (Chicago, IL, USA). A P value of 0.05 was considered significant.

## Results

**Figure 1:**
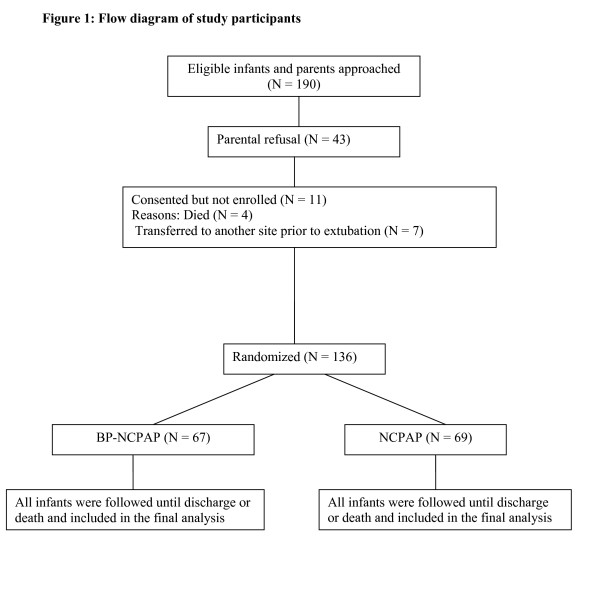
**Flow diagram of study participants**.

Of the 534 infants ≤ 1,250 grams admitted to the NICU during the study period, 348 infants were eligible for the study. Parents of 190 infants were approached of whom 43 declined and 147 consented. Four infants died and 7 were transferred to another site prior to randomization. Thus, a total of 136 neonates were enrolled (Figure 1). The trial had to be stopped prematurely prior to enrolment of the intended sample size due to a change in clinical practice in our unit that resulted in fewer infants being intubated from birth. Results are presented for the recruited subjects. No interim analysis was performed.

The demographic characteristics (birth weight, gestational age and sex) did not differ between participants and non-participants. Sixty-seven infants were randomized to BP-NCPAP and 69 to NCPAP. Baseline maternal and neonatal characteristics of the participants are presented in Tables 2 and 3, respectively. There were no significant differences between the two groups.

**Table 2:** Baseline maternal characteristics

Variable*	BP-NCPAP (N = 63)	NCPAP (N = 65)	P value
Maternal age (years) [Mean (SD)]	31.5 (6.3)	30.8 (5.9)	0.55

Gravida [Median (IQR)]	2 (1, 3)	2 (1, 3)	0.89

Parity [Median (IQR)]	0 (0, 1)	0 (0, 1)	0.97

Pregnancy induced hypertension/eclampsia [N (%)]	13 (21%)	17 (26%)	0.46

Essential hypertension [N (%)]	8 (13%)	8 (12%)	0.89

Prolonged rupture of membranes [N (%)]	20 (32%)	25 (38%)	0.42

Chorioamnionitis [N (%)]			
Clinical	9 (14%)	15 (23%)	0.20
Histological	29 (52%)	26 (43%)	0.32

Antenatal steroid [N (%)]			
Complete course	42 (67%)	47 (72%)	0.35
Partial course	11 (17%)	13 (20%)	
None	10 (16%)	5 (8%)	

*IQR = Inter-quartile range, N = Number, % = Percent, SD = Standard deviation

**Table 3:** Baseline characteristics of the study participants

Variable*	BP-NCPAP (N = 67)	NCPAP (N = 69)	P value
Gestational age (weeks) [Mean (SD)]	27.3 ± 1.9	27.4 ± 1.7	0.60

Birth weight (grams) [Mean (SD)]	901 ± 200	896 ± 156	0.86

Male [N (%)]	39 (58%)	31 (44%)	0.13

Apgar score (1 minute) [Median (IQR)]	5 (3, 7)	5 (2, 7)	0.56

Apgar score (5 minutes) [Median (IQR)]	8 (7, 9)	8 (7, 9)	0.63

Mode of ventilation [N (%)]			
IPPV	50 (75%)	51 (74%)	0.85
HFOV	14 (21%)	16 (23%)	
HFJV	3 (4%)	2 (3%)	

Age at first extubation (days) [Median (IQR)]	3 (1-67)	3 (1-62)	0.85

Time of blood gas prior to extubation (hours) [Median (IQR)]	9 (5, 14)	7 (5, 12)	0.15

Blood gas prior to extubation [Mean (SD)]			

pH	7.3 ± 0.1	7.3 ± 0.1	0.12

PCO_2_	47.1 ± 11.0	48.5 ± 10.9	0.48

FiO_2 _[Mean (SD)]	0.24 ± 0.06	0.26 ± 0.08	0.40

Fulfilled preset extubation criteria [N (%)]	47 (70%)	55 (80%)	0.20

Accidental extubation in infants who did not meet preset extubation criteria [N (%)]	6 (9%)	7 (10%)	0.98

Surfactant administration [N (%)]	61 (91%)	66 (96%)	0.32

Caffeine administration [N (%)]	65 (97%)	64 (92%)	0.44

*FiO_2 _= Fraction of inspired oxygen, HFJV = High frequency jet ventilation, HFOV = High frequency oscillatory ventilation, IPPV = Intermittent positive pressure ventilation, IQR = Inter-quartile range, N = Number, % = Percent, SD = Standard deviation

The incidence of sustained extubation following the first extubation was 67% (45/67) in the BP-NCPAP group compared with 58% (40/69) in the NCPAP group (P = 0.27). The reasons and time for reintubation in the first 7 days following extubation are presented in Table 4. The incidence of adverse events and short-term neonatal outcomes were similar between groups except for ROP which was higher in the BP-NCPAP group (P = 0.02) (Table 5). No infant developed pneumothorax following extubation with either mode.

**Table 4:** Comparison of primary outcome and extubation characteristics

Variable*	BP-NCPAP (N = 67)	NCPAP (N = 69)	P value
Successful extubation [N (%)]	45 (67%)	40 (58%)	0.27

Time of blood gas after extubation (hours) [Median (IQR)]	4 (2, 6)	2 (2, 4)	0.02

Blood gas after extubation [Mean (SD)]			
pH	7.4 ± 0.1	7.3 ± 0.1	0.02
PCO_2_	45.8 ± 13.4	47.8 ± 13.3	0.39

FiO_2 _[Mean (SD)]	0.27 ± 0.09	0.26 ± 0.06	0.30

Time to reintubation (days) [Median (IQR)]	2 (1, 4)	1 (1, 5)	0.76

Number and reasons for reintubation [N (%)]	N = 22	N = 29	
Hypercapnia	0	0	
FiO_2 _requirements > 60%	2 (9%)	0	0.14
Severe apnea defined as need for positive pressureventilation or frequent apnea defined as ≥ 4 minor apneic episodes per hour requiring moderate stimulation	13 (59%)	23 (79%)	
Combination of the above	7 (32%)	6 (21%)	

*FiO_2 _= Fraction of inspired oxygen, IQR = Inter-quartile range, N = Number, % = Percent, SD = Standard deviation

**Table 5:** Incidence of adverse events and short-term neonatal outcomes

Variable*	BP-NCPAP (N = 67)	NCPAP (N = 69)	P value
**Adverse events**			

Nasal septum breakdown [N (%)]	6 (8.9%)	9 (13%)	0.59

Eyelid edema [N (%)]	3 (4.5%)	2 (2.8%)	0.68

Feeding intolerance [N (%)]	8 (11.9%)	17 (25%)	0.08

Abdominal distension [N (%)]	16 (23.8%)	8 (12%)	0.07

Pneumothorax [N (%)]	0	0	

**Short-term neonatal outcomes**			

Mortality [N (%)]	3 (4%)	5 (7%)	0.49

New-onset sepsis after extubation [N (%)]	8 (12%)	5 (7%)	0.35

Bronchopulmonary dysplasia (oxygen dependency at 36 weeks PMA) [N (%)]	21 (31.3%)	22 (31.8%)	1.0

Necrotising enterocolitis [N (%)]	7 (10.4%)	7 (10.1%)	0.95

Grade 3/4 IVH/PVL	1/62 (1.6%)	5/65(7.7%)	0.41

ROP > stage 2 [N (%)]	11 (17%)	3 (5%)	0.02

Patent ductus arteriosus [N (%)]	36 (53.7%)	35 (51%)	0.82

*IVH = Intraventricular hemorrhage, N = Number, % = Percent, PVL = Periventricular leucomalacia, ROP = Retinopathy of prematurity

Multivariate regression analysis identified that birth weight was the most important predictor of sustained extubation (P = 0.003) regardless of the mode of CPAP used (Table 6). For every 100 grams increase in birth weight the odds of a successful outcome was 1.49 times higher.

**Table 6:** Predictors of successful extubation

Variable*	Odds Ratio (95% CI)	P value
Mode of CPAP	1.51 (0.71, 3.20)	0.28

Birth weight in increments of 100 grams	1.49 (1.11, 1.82)	0.003

Female	1.84 (0.87, 3.92)	0.11

Antenatal steroids	1.01 (0.98, 1.05)	0.47

Age at time of first extubation	0.31 (0.08, 1.32)	0.11

Accidental extubation	0.81 (0.30, 2.14)	0.67

* CPAP = Continuous positive airway pressure, CI = Confidence interval

## Discussion

In our study, we were unable to demonstrate the effectiveness of BP-NCPAP in facilitating sustained extubation. Further, the incidence of adverse events, reasons for reintubation and short-term neonatal morbidities except for ROP were similar in both groups. There may be several reasons for our inability to show a difference in the primary outcome. Firstly, we were unable to recruit the predetermined sample size to demonstrate a difference due to increasing use of non-invasive ventilation from birth. This is a major flaw of our study. Secondly, the overall rate of sustained extubation in our infants was much lower than anticipated. The rate of sustained extubation in our study ranged from 58% to 67% compared to a rate of 85% used to determine our sample size. Using these revised rates of sustained extubation and an effect size of 10%, we would need to recruit a total of 870 infants to demonstrate a difference. Obviously conducting such a trial requires a collaborative effort and is a major undertaking.

Our *a priori *hypothesis was to demonstrate/achieve a clinically significant increase in the rate of sustained extubation of 10% with the use of BP-NCPAP. With our limited sample size we were able to demonstrate an increase in the rate of sustained extubation by 9% in the BP-NCPAP group even though this difference was not statistically significant. We cannot rule out the possibility that if we had indeed achieved our targeted sample size we may have been able shown a statistically significant difference. In clinical practice, this difference of 9% may be considered clinically significant as there is increasing trend of using non-invasive ventilation to avoid the consequences of mechanical ventilation.

To our knowledge, there are no previous published studies that have evaluated BP-NCPAP for facilitating successful sustained extubation following intubation and ventilation at birth, i.e., used as a secondary mode. The trial was initiated at our site when infants ≤ 1,250 grams were routinely intubated and ventilated at birth and administered prophylactic surfactant if ≤ 27 weeks gestation. Biphasic-NCPAP is considered a form of nasal intermittent positive pressure ventilation (NIPPV) and therefore we compared the results of our study to those of NIPPV when used as a secondary mode. In an updated Cochrane review in 2008, Davis et al. [19] compared NIPPV vs. CPAP for preterm infants after extubation. They demonstrated a reduction in extubation failure rate [relative risk (RR) 0.39; 95% confidence interval (CI) 0.16, 0.97)] with the use of synchronized NIPPV (data from 3 trials with N = 159) and concluded that it may potentially be a way of augmenting NCPAP when used to prevent extubation failure. Further evidence that NIPPV facilitates successful extubation comes from a recent randomized controlled trial by Moretti et al. [20] in which 94% (30/32) of infants in the NIPPV group were successfully extubated (defined as no reintubation within 3 days) compared with 61% (19/31) in the NCPAP group (P = 0.01). The details of the 4 published trials on NIPPV are presented in Table 7.

**Table 7:** Review of the literature on nasal intermittent positive pressure ventilation (NIPPV) vs. nasal continuous positive airway pressure (NCPAP) for preventing extubation failure

Study author, Year	Inclusion criteria	Intervention group	Control group	Primary outcome	Results
Barrington 2001	BW < 1,251 grams PNA < 6 wks	NSIMV (N = 27)	NCPAP (N = 27)	Extubation failure at 72 hours	4/27 (14%) vs. 12/27 (44%) in the NSIMV vs. NCPAP (P < 0.05) Median age at extubation (range) 3 (1, 29) vs. 3 (1, 40) days

Friedlich 1999	BW 500-1,500 grams	NP-SIMV (N = 22)	NCPAP (N = 19)	Respiratory failure at 48 hours	1/22 (5%) vs.7/19 (37%) in the NP-SIMV vs. NCPAP group (P = 0.016) Median age at extubation (range) 18.5 (1, 120) vs. 21(1, 54) days

Khalaf 2001	GA ≤ 34 weeks, RDS	SNIPPV (N = 34)	NCPAP (N = 30)	Remained extubated at 72 hours	32/34 (94%) vs. 18/30 (60%) in the SNIPPV vs. NCPAP group (P < 0.01) Median age at extubation (range) 4 (1, 83) vs. 2.5 (1, 106) days

Morretti 2008	BW < 1,251 grams PNA < 14 days	NFSIPPV (N = 32)	NCPAP (N = 31)	Remained extubated at 72 hours	30/32 (94%) vs. 19/31(61%) in the NFSIPPV vs. NCPAP (P < 0.01) Median age at extubation (range) 4 (1, 14) vs. 6 (1, 14) days

*BW = Birth weight, CPAP = Continuous positive airway pressure, GA = Gestational age, NCPAP = Nasal continuous positive airway pressure, NSIMV = Nasal synchronized intermittent mandatory ventilation, NFSIPPV = Nasal flow synchronized intermittent positive pressure ventilation, PNA = Post natal age, SNIPPV = Synchronized nasal intermittent positive pressure ventilation

The above findings are in contrast to the results of our study. Possible explanations for the difference in the effectiveness of NIPPV compared to BP-NCPAP include variations in the: 1) definition of sustained extubation, 2) the median age of extubation and 3) ventilatory parameters used to prevent reintubation. The duration of successful extubation in the NIPPV trials was defined as 48 hours [21] to 72 hours [20,22,23] vs. 7 days in our study. Further, the median age of extubation was day 3 in our study vs. 7 days [22,23] and 18.5-21 days [21] in the NIPPV studies. This later age of extubation may have resulted in resolution of co-morbidities such as a clinically significant patent ductus arteriosus in the first 7-10 days, which could contribute to successful sustained extubation.

**Figure 2:**
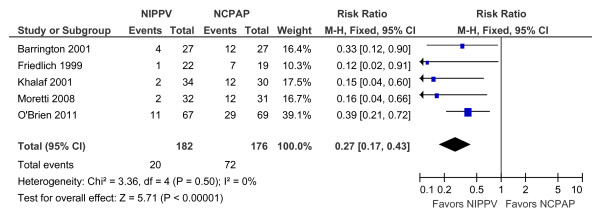
**Comparison of the effectiveness of NIPPV vs. NCPAP to prevent extubation failure**.

As BP-NCPAP is considered to be a form of NIPPV, we conducted a meta-analysis including data from the 4 published studies and our results (Figure 2). The incidence of extubation failure was lower with the use of NIPPV and BP-NCPAP compared to NCPAP [Relative risk (RR), 0.27; 95% confidence interval (CI), 0.17, 0.43; P < 0.01]. No significant statistical heterogeneity was noted for this outcome. The risk difference was -0.30, 95% CI (-0.38, -0.21; P < 0.01). The number needed to prevent one infant from being reintubated was 3 (95% CI, 2, 5).

When we designed our study, there were no previous studies to guide us in our choice of ventilatory parameters to be used to provide effective BP-NCPAP. The maximum upper level of CPAP that can be set with BP-NCPAP is less than that used for NIPPV and with the use of endotracheal tube and ventilation. In our study, the upper level of CPAP varied from 8 to 10 cmH_2_O based on the oxygen requirements of the infant. The inspiratory and expiratory time were set at 1.0 and 2.0 seconds respectively resulting in a pressure exchange rate of 20 breaths per minute (cycle/minute at upper level). We were unable to synchronize the delivery of the upper level of CPAP with this device. The combination of lack of synchronization with low pressure exchange rate and upper level of CPAP may have been critical factors contributing to our failure in facilitating sustained extubation. With most modes of NCPAP or indeed NIPPV synchronisation is imperfect, using either a Graseby capsule on the infant's abdomen or most recently a flow detector at the nares [24]. When a higher rate of ventilation is used as in most modes of NIPPV then synchronisation happens more often just by chance. Synchronisation may be important for entrainment of tidal volumes both on inspiration and expiration [24-26] and that this may in part explain the clinical benefit of synchronised NIPPV.

Recently, BP-NCPAP has been evaluated for infants with moderate respiratory distress syndrome as a primary mode of ventilation. Infants between 28-34 weeks gestation were randomized to either BP-NCPAP or NCPAP in the first hour of birth. The use of BP-NCPAP was associated with shorter respiratory support and oxygen dependency with no difference in the rate of reintubation [27]. In the BP-NCPAP group; the investigators used a pressure exchange rate of 30 bpm, inspiratory time of 0.5-0.7 seconds and upper and lower CPAP level of 8 and 4.5 cm H_2_O respectively.

Adverse events such as increased risk of pneumothorax, nasal septal trauma, feeding intolerance, abdominal distension and gram-negative sepsis secondary to nasal mucosal barrier breakdown have been described in the literature with the use of various forms of NCPAP [5,28,29]. The incidence of pneumothorax was reported to be 6.8% [5] and 9% [29] respectively in the Support and the COIN trial where NCPAP was used soon after birth. We did not find any increase in the risk of pneumothorax in our trial. One major difference between the previous studies and ours is that we used NCPAP after the first extubation rather than using it as a primary mode of ventilation. Increased risk of gastric perforation has been reported with the use of NIPPV [30]. No differences in the rate of short-term neonatal morbidities, especially BPD were noted between groups in our study. We did find an increased risk of ROP in the BP-NCPAP compared to NCPAP group (P = 0.02). It is important to recognize that our study was not powered to detect differences in these secondary outcomes so the finding of increased incidence of ROP needs to be confirmed/refuted in future studies.

## Conclusion

BP-NCPAP may be used safely and effectively to assist in weaning from mechanical ventilation. However, the effectiveness and safety of BP-NCPAP compared to NCPAP needs to be confirmed in a large multi-center trial as our study conclusions are limited by inadequate sample size.

## Competing interests

The authors declare that they have no competing interests.

## Authors' contributions

KOB conceived the study, participated in designing the study, assisted in data collection and participated in the checking, analysis and interpretation of the data and drafting the manuscript. CC conceived the study, participated in designing the study, recruited patients, assisted in data collection and in the checking, analyzing and interpretation of the data, and drafting of the manuscript. Both LH and LW participated in the recruitment of study patients, collected data and had input in drafting of the manuscript. VS contributed to the design of the study, participated in the checking, analysis and interpretation of the data and in drafting of the manuscript. All authors have read and approved the final manuscript.

## Pre-publication history

The pre-publication history for this paper can be accessed here:

http://www.biomedcentral.com/1471-2431/12/43/prepub
